# Complete genome sequence of tulip virus X, a Korean isolate from *Tulipa gesneriana*

**DOI:** 10.1128/mra.00948-23

**Published:** 2023-12-20

**Authors:** Sangmin Bak, Hyowon Jeong, Su-Jin Son, Minseok Kim, Taegun Lim, Su-Heon Lee

**Affiliations:** 1 School of Applied Biosciences, Kyungpook National University, Daegu, Republic of Korea; 2 Department of Plant Protection and Quarantine, Kyungpook National University, Daegu, Republic of Korea; 3 Agricultural Technology Center, Dalseong-gun Office, Daegu, Republic of Korea; 4 Department of Applied Biology, Kyungpook National University, Daegu, Republic of Korea; 5 Department of Plant Medicine, Kyungpook National University, Daegu, Republic of Korea; 6 Institute of Plant Medicine, Kyungpook National University, Daegu, Republic of Korea; Katholieke Universiteit Leuven, Leuven, Belgium

**Keywords:** tulip virus X, tulip, high-throughput sequencing, complete genome sequence

## Abstract

In this study, the presence of tulip virus X (TVX) in Korean tulips was confirmed through high-throughput RNA sequencing. Its complete genome sequence of 6,056 nucleotides was determined via Sanger sequencing, exhibiting a 99.24% nucleotide identity with TVX-J isolate. This signifies a previously unreported presence of TVX outside Japan.

## ANNOUNCEMENT

Tulips, members of the family Liliaceae, are popular ornamental plants known for their diverse colors and shapes. Several viruses were reported in recent virome analysis of tulip plants (1, 2). Tulip virus X (TVX) is a member of the genus *Potexvirus*, which belongs to the family *Alphaflexiviridae*. Since the initial report of the complete genome sequence (CGS) in Japanese tulips (3), no further reports emerged until the genome of viola mottle virus was confirmed identical to that of TVX (4).

In April 2021, 86 tulip plants exhibiting viral symptoms in leaves (mosaic, yellowing, and malformation) and flowers (color breaking) were collected in five regions of Korea: Chilgok (35°54′48.5″N 128°24′35.6″E), Chuncheon (37°43′38.7″N 127°42′10.4″E), Goseong (34°59′42.6″N 128°19′40.1″E), Yecheon (36°44′00.6″N 128°26′02.4″E), and Yesan (36°41′09.4″N 126°48′31.7″E). Total RNA was extracted from pooled samples using the Maxwell 16 LEV Plant RNA Kit (Promega, USA). Subsequently, a single library was constructed using the TruSeq Stranded Total RNA LT Sample Prep Kit for Plants (Illumina, USA) and subjected to 100-bp paired-end sequencing on an Illumina NovaSeq 6000 (Macrogen, Korea). A total of 628 million reads were generated out of 641 million using Trimmomatic version 0.38 and *de novo* assembled into 498,795 contigs using Trinity version trinityrnaseq_r20140717 with default parameters. These contigs were then subjected a BLASTn with an e-value cut-off of 10^−5^, against the NCBI nucleotide database version 20180116, leading to the identification of contigs related to lily symptomless virus (LSV), olive mild mosaic virus (OMMV), tulip breaking virus (TBV), and TVX. Confirmed through reverse transcription-PCR (RT-PCR) assays (1, 2), individual samples exhibited sole infections: five LSV, eight TBV, nine TVX, and seven OMMV. Co-infections included two LSV + TVX, four LSV + OMMV, two TBV + TVX, twelve TBV + OMMV, and four LSV + TBV + OMMV. Total RNA was extracted from a TVX-positive sample using the easy-spin Total RNA Extraction Kit (iNtRON, Korea) and used for cDNA synthesis with Oligo d(T) utilizing the SuperiorScript III cDNA synthesis kit (Enzynomics, Korea). PCRs were performed using 10 primer pairs designed to have a 100- to 200-bp overlap at both ends (Table 1). The amplicons were cloned using the All-in-One Cloning Kit (BioFact, Korea) and sequenced using the ABI 3730XL DNA Analyzer (Bioneer, Korea). Subsequently, the sequencing reads underwent quality trimming with a Phred cut-off score of ≥20. The sequences of both ends of genome were determined using 5′/3′ RACE System for Rapid Amplification of cDNA Ends (Thermo Fisher Scientific, USA) with gene-specific primers (Table 1). The assembly of the obtained fragments was performed using UGENE version 44.0 (Unipro, Russia).

**TABLE 1:** Primer list used to determine the complete genome sequence for Korean isolate of tulip virus X

Fragment^*a*^	Primer name	Primer sequence (5′ to 3′)	Location
1	TVX-F74	CTAAGAAGCTAGGTAAACGAC	74–94
	TVX-R816	CGTTGGGCAGTGACAGTG	799–816
2	TVX-F673	CTACACGCTTACCTACTCC	673–691
	TVX-R1421	GACTTTGGTTGGCGCAAGC	1403–1421
3	TVX-F1261	GATCAAGGCTCTTCAGTGG	1261–1279
	TVX-R2096	GCAGCACAACTTCGACTGG	2078–2096
4	TVX-F1834	GCAACTCAACAGAAGCCCC	1834–1852
	TVX-R2635	CATGGTGGTGTCGCTGTC	2618–2635
5	TVX-F2520	CATTGGTGAAGAAAGCGGC	2520–2538
	TVX-R3418	GTACCTCCGCATGTATCTG	3400–3418
6	TVX-F3211	CAAGTCCCTCCAACAGATC	3211–3229
	TVX-R4114	GTGTGCTTGTTCAGGGGAG	4096–4114
7	TVX-F3950	GATCCCCTAAAGCTTCACAG	3950–3969
	TVX-R4730	GACACTCTCTGGGCAATGG	4712–4730
8	TVX-F4514	CTTCCTCAAGACCACCAGC	4514–4532
	TVX-R5253	CTTGTTAGAAGTTCGGGCG	5235–5253
9	TVX-F5099	GACTCATTCTACTTCTGTCTC	5099–5119
	TVX-R5788	CGTTGGCTGTTATTTCGTCG	5769–5788
10	TVX-F5557	GTCGTCAAAGTCTCAACCAC	5557–5576
	TVX-F5946	CTGGGTTCGCAGCTGTAG	5929–5946
5′-termini^*b*^	TVX-RR185	CTTTCTTCATCACTCGGTAGG	165–185
	TVX-RR285	GTTATGGCAAGTGGGTGTG	267–285
3′-termini^*b*^	TVX-RF5769	CGACGAAATAACAGCCAACG	5769–5788
	TVX-RF5871	CCAGCTCTCTAACACCTCC	5871–5889

^*a*^ Each fragment was designed to have an overlap of approximately 100–200 bp on both ends of the amplification product.

^*b*^ Indicates gene-specific primers used for rapid amplification of cDNA ends to determine both ends of the complete genome sequence.

**Fig 1:**
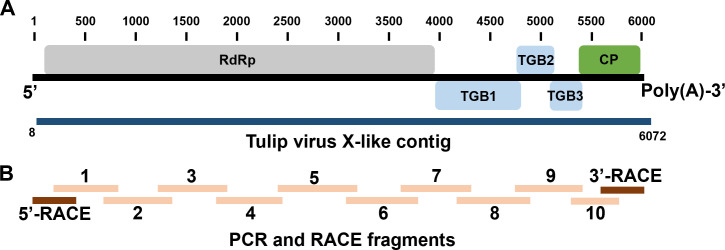
Genome schematic of the Korean isolate of TVX. (**A**) The schematic represents the genome organization of the Korean isolate of TVX. The genome contains five open-reading frames (ORF): ORF1 (RNA-dependent RNA polymerase, RdRp; nt 96–4181), ORF2 (triple gene block1, TGB1; nt 4183–4875), ORF3 (triple gene block2, TGB2; nt 4841–5170), ORF4 (triple gene block3, TGB3; nt 5007–5291), and ORF5 (coat protein, CP; nt 5303–5926). The blue bar indicates the tulip virus X-like contig, which was composed of 6,076 nt and assembled *de novo* from 24,068 reads. (**B**) The positions of amplified fragments for determining the complete genome sequence of the TVX Korean isolate.

The determined genome was composed of 6,056 nucleotides, with a GC content of 57.15% (Fig. 1). Five open-reading frames (ORFs) were predicted using the NCBI ORF finder with default parameters. The CGS exhibits 99.24% nucleotide identity to TVX-J (GenBank AB066288) with 100% query coverage in NCBI BLASTn. To our knowledge, there have been no previous cases of TVX reported in any natural hosts in Korea. Given the exclusive dependence on imported bulbs in Korean tulip cultivation, these findings imply a potential introduction of TVX through imported bulbs.

## Data Availability

The complete genome sequence of tulip virus X, a Korean isolate, was deposited in GenBank under the accession number OP856636 .1. The original data from both high-throughput sequencing and Sanger sequencing, used for establishing the complete genome sequence of Korean TVX, have been archived in the Sequence Read Archive under the accession numbers SRR24725337 and SRR26283619, respectively.

## References

[1] Bak S , Kim M , Kim HJ , Lee HK , Kwon M , Hong JS , Min DJ , Byun HS , Lee JS , Song JK , Nam KH , Lee SH . 2023. First report of tulip virus X infecting tulip (Tulipa gesneriana) in Korea. Plant Disease 107:238. doi:10.1094/PDIS-12-21-2762-PDN

[2] Bak S , Kim M , Kim HJ , Kang EH , Kang DH , Min JG , Han S , Lee HK , Lee SH . 2023. First report of olive mild mosaic virus in imported tulips (Tulipa gesneriana) in Korea. Plant Dis 107:2895. doi:10.1094/PDIS-03-23-0527-PDN

[3] Yamaji Y , Kagiwada S , Nakabayashi H , Ugaki M , Namba S . 2001. Complete nucleotide sequence of tulip virus X (TVX-J): the border between species and strains within the genus Potexvirus. Arch Virol 146:2309–2320. doi:10.1007/s007050170004 11811681

[4] Matsumoto O , Miyazaki A , Tokoshima J , Suzuki T , Yoshida T , Okano Y , Nijo T , Maejima K , Namba S , Yamaji Y . 2021. Complete genome sequence of viola mottle virus, revealing its synonymous relationship to tulip virus X. Arch Virol 166:2343–2346. doi:10.1007/s00705-021-05129-4 34097143

